# Editorial: The Notion of the Native Speaker Put to the Test: Recent Research Advances

**DOI:** 10.3389/fpsyg.2022.875740

**Published:** 2022-03-29

**Authors:** Mila Vulchanova, Valentin Vulchanov, Antonella Sorace, Cristina Suarez-Gomez, Pedro Guijarro-Fuentes

**Affiliations:** ^1^Norwegian University of Science and Technology, Trondheim, Norway; ^2^University of Edinburgh, Edinburgh, United Kingdom; ^3^Department of Spanish, Modern and Classic Philology, University of the Balearic Islands, Palma, Spain

**Keywords:** native speaker, language competence, second language acquisition, psycholinguistics, sociolinguistics and bilingualism

## Introduction

The notion of the native speaker has occupied a prominent place in foreign/second language research and theoretical linguistics: it has influenced both the way we theorize about language and the way we conduct empirical research, and has had practical implications concerning second language pedagogy. In the second language tradition, it was needed as a norm and a standard to evaluate L2 attainment, and, as such, has functioned as a benchmark in terms of goals for L2 instruction (Davies, 2003). In line with this tradition, second language and lingua franca speakers' achievements have ideally been compared with those of monolingual native speakers, although these constitute different groups of speakers, with different needs and abilities. In a similar vein, much psycholinguistic and bilingualism research uses the “native speaker” norm, based on an idealized first language (L1) competence, adopting the inclusion of a control group of monolingual speakers of the language as default in empirical research.

The idealization of the native speaker has its roots in theoretical linguistics, in all likelihood arising from Bloomfield (1927) stringent criteria, whereby the native speaker is an idealized bearer of L1 competence, thus downplaying individual variation. On Chomsky's early definition (Chomsky, 1965), native speakers are characterized by the ability to provide valid judgments on their language and identify ill-formed grammatical expressions in that language, although they may not be able to explain exactly why they are ill-formed. Other attempts at characterizing the native speaker resort to specific key abilities, such as saying the same thing in different ways, hesitating and using fillers, predicting what the other person is going to say, and adding new verbal skills from mere language experience, such as immersion (Halliday, 1978).

Current understanding has moved away from this idealized characterization of the native/monolingual speaker. One of the main reasons for this is the recognition of the amply documented individual variation in language competence, both in adult native speakers, and across language development (Bates et al., 1995; Dabrowska, 2014; Tomblin and Nippold, 2014). Furthermore, neuroscience research has documented the absence of structural differences in key brain structures underlying language use in monolinguals and bilinguals with a language acquisition onset before 3 years of age (Klein et al., 2013). In addition, the role of age of onset as key factor in language competence, has been confirmed in a recent study (Bylund et al., 2020). We also have evidence that L2 speakers may attain high levels of proficiency even with a late onset (Sorace and Filiaci, 2006 on “near-native speakers”; Abrahamsson and Hyltenstam, 2009), and can display sensitivity to properties of the L2, including prediction of upcoming words, in response to mere exposure, in a way similar to monolingual L1 speakers (Treffers-Daller and Calude, 2015). It has also been shown that variation in certain cognitive abilities and competencies can account for exceptional skills at second language learning (Vulchanova et al., 2012a,b; Hyltenstam et al., 2018). Cook (1996), for instance, proposes to replace the notion of ideal speaker with “multi-competent language user.” Global varieties of the same language offer systematic differences at all levels of language structure, and further challenge unitary perspectives on a single native speaker standard. Emerging new fields of research, such as heritage language and language attrition, are further challenging earlier perceptions of how native speakers should be defined and are re-defining previous assumptions in the field.

## The Evidence

One of the reasons for revising the notion of the native speaker has been the *multilingual turn* (May, 2014), which has given rise to heightened awareness of the cognitive and neuro-biological consequences of being exposed to more than one language over the lifetime (Kroll and Bialystok, 2013). Another key factor has been mounting evidence and empirical findings suggesting large inter-individual variation in native speakers of specific languages as well as the recognition of different sub-groups or populations which all can be characterized as “native speakers.”

### Individual Variation

Individual variation in language competence has been amply documented, both in adult native speakers, and across language development (Bates et al., 1995; Mulder and Hulstijn, 2011; Dabrowska, 2012; Tomblin and Nippold, 2014). Thus, Mulder and Hulstijn (2011) provide evidence of variation in both lexical and production skills in a sample of adult native speakers of Dutch as a function of age and level of education and profession. In a similar vein, Street and Dabrowska (2010) provide evidence of variability in quantifier knowledge and competence in the comprehension of passives among young adult native speakers of English contingent on education level. Dabrowska (2012) provides a comprehensive review and discussion of findings in the field of native speaker knowledge in a variety of domains, including inflectional morphology, passives, quantifiers, and syntax. A possible account may be sought in how language learners attend to, and interact with, the input and eventually end up with different grammars, while other differences may be attributed to more varied linguistic experience as a result of education. On this backdrop, Dabrowska (2012) suggests that such findings have consequences for research on bilingualism, ultimate attainment in second language acquisition, as well as important methodological implications for the science of language. Inter- and intra-individual variability in adult native speakers of German is documented in the contributions to the current Research Topic. Shadrova et al. show that the variation in certain linguistic aspects in corpus native speaker data undermines general statements about quantitative expectations in L1. The authors also find differences between the phenomena under study. Thus, while morphological and syntactic sub-classes of verbs and nouns show great variability in their distribution in native speaker writing, other, coarser categories, like parts of speech, or types of syntactic dependencies, behave more predictably and homogeneously.

Children acquiring language are another group of native speakers where large variation can be observed applying to expected language competence at specific ages. Research has documented that milestones in language development are less related to age than to earlier acquired language skills (as well as non-linguistic skills) which tend to scaffold them. This may explain the large variation in early vocabulary size and the dependence of early grammar skills on lexical skills in early development (Bates et al., 1995; Bates and Goodman, 1997). Fenson et al. (1994) provide evidence of extensive variability in the rate of lexical, gestural, and grammatical development in a large sample of infants between 8 and 30 months of age. This variability in both the onset and the course of development challenges the notion of a “model” child. At the same time, however, reliable intercorrelations can be found both concurrently and predictively between specific communication skills indicative of an “internal” causality mechanism, with development contingent on an underlying trajectory.

The evidence from typical language development and its “internal” logic is further confirmed by research on neuro-developmental deficits, such as autism and language impairment. Thus, deictic gestures have been documented as an important and reliable predictor of language status for infants between 15 and 20 months (Colonnesi et al., 2010). However, no relationship can be established later, as other communication skills which are more directly related to language take over as predictors. In contrast, for infants and children with autism, pointing gestures continue to exert a strong predictive relationship also at later stages, as shown by Ramos-Cabo et al. (2022). Neuro-developmental deficits also highlight the fact that not all children, who, on other criteria may be considered native speakers, achieve uniform ultimate attainment in the first language, either as a result of impairment in the mechanisms which underlie language acquisition, such as the phonological loop of the memory system or impairment in the mechanisms which ensure efficient language use, e.g., phonological processing problems or attention deficits (Bishop, 2009).

### Brain and Age

Neuroscience research provides further evidence challenging the assumed differences between monolingual native speakers and bilinguals exposed to more than one language during the first years of life. Thus, Klein et al. (2013), document that there are no structural differences in key brain structures underlying language use in monolinguals and bilinguals with a language acquisition onset before 3 years of age. The role of age of onset as key factor in language outcomes has been systematically confirmed (Bylund et al., 2020). Recent mounting evidence from bilingual children suggests incomplete acquisition or even loss (Ventureyra, 2004) when exposure to input is terminated before ultimate attainment. There is also evidence of an advantage in processing or learning later in life the language which individuals were originally exposed to in early infancy, e.g., in the case of internationally adopted children for whom exposure to the birth language has been discontinued (Hyltenstam et al., 2009; Oh et al., 2010). Interestingly, the birth language of internationally adopted children leaves a trace in the neural organization of their language system, and research documents influence of early experience on later brain outcomes. In a study of internationally adopted children (aged 9–17 years), who were completely separated from their birth language (Chinese) at 12.8 months of age, Pierce et al. (2014) show, that, on average, these children displayed brain activation to Chinese linguistic elements that precisely matched that of native Chinese speakers, despite the fact that the international adoptees had no subsequent exposure to Chinese and no conscious recollection of that language. Crucially, no similar activation was found in a control (monolingual) group of speakers of French, the first language of the adopted children. Such findings further raise questions about the nature of, and criteria on which we identify, native speakers. This study and other studies of children with delayed or qualitatively compromised input (e.g., children born profoundly deaf and exposed to oral language following cochlear implantation and internationally adopted children who have delayed exposure to the adoption language; children who experience impoverished language input, that is, children who experience early bouts of otitis media and signing deaf children born to non-signing hearing parents) demonstrate that language outcomes in a first language are the result of an intricate and dynamic interaction of a number of factors, some internal to the child (e.g., phonological working memory) and external factors (e.g., the quantity and quality of the input) (Pierce et al., 2017).

### First and Second Language Speakers

Evidence from the SLA research has further questioned the notion of the native speaker. Thus, some advanced second language speakers with a late onset have been shown to perform similarly to participants with native speaker competence (Abrahamsson and Hyltenstam, 2009), although not in all respects: some grammatical properties requiring “interfacing” conditions of a non-linguistic nature remain variable in very advanced levels of L2 competence (Sorace and Filiaci, 2006; Tsimpli and Sorace, 2006; Sorace, 2011). In addition, sensitivity to properties of the target language (an L2 for participants in that research), such as, e.g., prediction of upcoming words and language structure has been documented in L2 learners in response to exposure, in a way similar to monolingual L1 speakers (Treffers-Daller and Calude, 2015). The contribution by Hidalga et al. explores the factors which may facilitate the acquisition of another language to proficiency levels comparable with monolingual native competence. In an EEG experiment, they show that grammar phenomena which are shared by the two languages of early and proficient bilinguals are more likely to be acquired to native level and elicit similar brain responses in both groups of speakers. Furthermore, variation in certain cognitive abilities and competences have been found to account for exceptional skills at second language learning (Hyltenstam et al., 2018). Vulchanova et al. (2012a) document German language skills similar to age-matched children acquiring German as a native language in a child with exceptional skills at learning foreign languages, who learned German from mere exposure to a German TV channel.

The prevalence of multilingualism on a global level has also given impetus to re-conceptualisations of the notion of the native speaker. Cook (1996, 1999) proposes to replace the notion of “ideal speaker” by the notion of “multi-competent language user.” In addition, global varieties of the same language (such as e.g., English) offer systematic differences at all levels of language structure, and further challenge unitary perspectives on a single native speaker standard. A similar concept, from the field of English as a Lingua Franca (ELF), is that of “similect,” coined by (Mauranen, 2012: 29), to refer to a linguistic variety spoken by people with different L1s, with features transferred from the L1 by individual speakers.

In the field of World Englishes, second language varieties which emerged after a process of language contact between English and the relevant local languages (e.g., Indian English, Jamaican Englishes, etc.) are also relevant in this respect, where the concept of native speaker also needs to be recontextualized. Here, the traditional concept of the native speaker as an idealized monolingual speaker has to be adapted to these multilingual contexts and speakers who speak native varieties which differ from what has traditionally been considered the norm (Mesthrie, 2010). These native varieties have been shaped after a second language acquisition process in language contact contexts and in a globalized world, and determined by linguistic forces such as the increasing use of isomorphic structures which aim at communicative efficiency.

### Language Attrition, Heritage Languages, Signers

Defining the native speaker is even more problematic in the face of language attrition and the increase in number of heritage language speakers. A distinction has to be made between attrition in first-generation L1 speakers and inter-generational attrition in heritage speakers, and the connection between the two is currently being explored (see, e.g., Sorace, 2016). A change in native language competence as a result of decreased exposure to the first language is increasingly becoming common in a global world with increased population mobility. Language attrition is manifested at all levels and has been documented particularly at the lexical and syntactic levels (see Schmid and Köpke, 2019), but can also be manifested at the level of processing (see Roman and Gómez-Gómez's contribution in this special issue), and phonology. In their contribution, Kornder and Mennen provide evidence that native speakers of Austrian German who have resided over a long period in and English-speaking environment sound less native to naïve Austrian German monolingual judges. Interestingly, however, this judgement differed significantly from that of a group of naïve Austrian German-English bilinguals, who rated the target group as more native-sounding.

The contribution by Wiese et al. investigated heritage Greek, Russian, Turkish, and German in comparison to monolingual, non-heritage speakers and found non-canonical patterns not only in bilingual, but also in monolingual speakers, including patterns that have so far been considered absent from native grammars, in domains of morphology, syntax, intonation, and pragmatics. This study also confirms other findings of monolingual heterogeneity and reports a degree of lexical and morphosyntactic inter-speaker variability in monolinguals, sometimes higher than that of bilinguals, further challenging the model of the streamlined native speaker. Also, in some respects, the monolingual participants and the heritage speakers performed similarly.

Tsehaye et al. address further problems with applying the notion of the native speaker in the context of heritage speakers. In this contribution to the Research Topic, they provide evidence by focusing both on similarities and differences between heritage speakers and monolingually-raised speakers, respectively, in their heritage and majority languages. Heritage speakers are an interesting case to study since they are bilinguals who acquire a family (heritage) language, often from parents who are undergoing attrition, and a societal (majority) language in early childhood. In such a way, naturalistic exposure from early childhood qualifies them as native speakers of their heritage language. In addition, some heritage speakers are simultaneous bilinguals, which makes them native speakers of their majority language as well. Others are early second language acquirers who may be indistinguishable from simultaneous bilinguals. Thus, heritage language, while being a challenge for traditional assumptions, also provides a suitable test-bed for these notions. It is clear that research on L1 attrition, within and across generations, flags up important issues in our understanding of the notion of “native speaker” not only of bilingualism and language learning, but also—more generally—in linguistics research. Data showing a convergence between L1 speakers' attrition and advanced L2 speakers' acquisition (Sorace, 2011, 2016) pave the way to the hypothesis that L1 changes may be functional to successful L2 learning: this further undermines the use of the monolingual native speaker as the point of reference, both in research and in society.

Further issues with the notion arise in the context of sign language users depending on the circumstances of acquisition. In their contribution to this Research Topic, Cheng et al. argue against applying the notion in sign language research and psycholinguistics, because it has been inconsistently conceptualized. In addition, factors, such as age, order, and context of acquisition, in addition to social/cultural identity, are often differentially conflated. Zorzi et al. argue further that, given that around 95% of deaf infants are born into a hearing family, deaf signers are exposed to a sign language at various moments of their life, and not only from birth. Since the linguistic input these children are exposed to is not always a fully-fledged natural sign language, the notion of native signer as someone exposed to language from birth cannot be applied. In their contribution, the authors present the results of the first large-scale cross-linguistic investigation on the effects of age of exposure to sign language in each of three sign languages (Catalan Sign Language, French Sign Language, and Italian Sign Language). This study shows the importance of exposure from birth as a “nativeness” criterion, in so far sign language is concerned, with significant differences across language and tests between signers exposed to sign language from birth and those exposed in the first years of life, at least for syntax. On the backdrop of their results across the three different groups, the authors further argue against the generalized use of native signer's grammar as the baseline for language description and language assessment.

## Contrasting Views

The evidence reviewed above indicates that questioning the notion of the native speaker has gained recognition from a variety of perspectives and fields, including the difficulties to define and operationalize it, its possible bias toward monolingualism, and its potential application to exclusionary purposes (Dewaele, 2018). The traditional definitions of what “first language” means is today at stake because of the evidence of numerous forms of initial bilingualism (Grosjean, 2010) and by the understanding that speakers themselves modify it (Seals, 2019). The negative consequences of applying native speaker standards have been observed also in second/foreign language pedagogy. For instance, *native-speakerism* (the adherence to an (idealized) monolingual target language standard) has been defined as:

“(…) a neoracist ideology that has wide-ranging impact on how teachers are perceived by each other and by their students. By labeling teachers as separate “native speakers” and “non-native speakers,” it falsely positions them as culturally superior and inferior with separate roles and attributes” (Holliday, 2018: 1).

Waddington (2021) provides evidence that the “ideal native speaker” model prevails in pre-service teacher assumptions and beliefs and that the latter not only serve to perpetuate the ideal itself, but also reinforce disempowering and discriminatory attitudes among the profession, which are both outdated and incongruent with current multilingualism inspired policies in early childhood education.

The notion of the native speaker has thus engendered contrastive, but not incompatible views. While some call for completely removing the notion from research, language theory and language pedagogy (see Dewaele, 2018 for an overview), others are proposing critical and well-argued re-conceptualisations. Among the first articulate proposals in that respect is Escudero and Sharwood Smith (2001). They propose a graded notion based on Rosh's prototype theory whereby some speakers, on specific criteria and under certain circumstances will count as native speakers. The criteria these authors propose are intra-linguistic (language competence proper) and extra-linguistic (initial and later language environment, education and literacy). The extra-linguistic aspect has been highlighted in Cook (1999) and Davies (2003) who suggest that language acquisition in naturalistic circumstances is one of the hallmarks of the concept of the native speaker. Importantly, Escudero and Sharwood Smith (2001) distinguish between applying those criteria by naïve (native) speakers of the language and linguists. Based on his research and applying the Shared/Basic Language Cognition framework, Hulstijn (2019) proposes two ways of defining native speakers in terms of language cognition: in terms of shared/basic language cognition and non-shared/extended/higher language cognition. He also proposes ways of defining native speakers in extralinguistic terms along (a) the biographical/ecological dimension of degrees of being bilingual and (b) the dimension of literacy. Thus, it is being argued that differences in native speakers' language cognition can be primarily conceptualized as a function of their memberships along the extralinguistic dimensions.

### New Speakers in Demo-Linguistics: Language Revitalization and Language Maintenance. Notion Still Useful?

Despite theoretical refinement and empirical evidence challenging the native speaker concept, it continues to be used, especially in socio- and demo-linguistics. Although its application may vary, language censuses and ethnolinguistic surveys often include it to refer to groups of people who acquired the same language(s) with their family of origin (Humbert et al., 2018). This perseverance could be probably explained because, in quantitative terms, the native speaker concept may still be applicable. Study after study corroborate that, in multilingual societies, there exists a strong correlation between the condition of being a first language speaker of a given language and scoring higher than speakers of other languages in terms of language proficiency, as well as from the point of view of language use, language dominance, and identification with the language. Needless to say, this strong association does not mean that native-speakerness is always the best predictor of linguistic performance, competence, or attitudes, nor does it allow for ecological fallacy, because the characteristics of individuals are most of the time not determined by the group they belong to.

Further evidence in defense of the notion comes from the fields of language revitalization (Hornsby, 2015; O'Rourke and Ramallo, 2015; Glinert, 2017), language survival and language maintenance, where a vibrant community of native speakers can safeguard and maintain a threatened language (Fishman, 1991; UNESCO, 2003; Phaidin and Cearnaigh, 2008; Giollagain, 2014a,b).

## Concluding Remarks

This Research Topic has aimed to engender a discussion of the notion of the native speaker, from a theoretical point of view, and informed by empirical findings.

Individual contributions introduce important methodological advances in the field by elaborating design and sample standards for future research in bilingualism, heritage language and language attrition (Duñabeitia and Carreiras, 2015; Paap et al., 2015). The current contributions are from the fields of psycholinguistics, sociolinguistics, second language learning, bilingualism, heritage languages, language attrition, and present diverse language learning and acquisition scenarios and languages/linguistic phenomena from different fields, thus encouraging interdisciplinary research. The evidence is rich, but not controversial. It is also unified in suggesting that a reconceptualization of the notion of the native speaker is mandatory for the purposes of language theory, empirical research and language second language instruction.

## Author Contributions

MV, VV, AS, CS-G, and PG-F wrote the manuscript and approved the final version. All authors contributed to the article and approved the submitted version.

## Conflict of Interest

The authors declare that the research was conducted in the absence of any commercial or financial relationships that could be construed as a potential conflict of interest.

## Publisher's Note

All claims expressed in this article are solely those of the authors and do not necessarily represent those of their affiliated organizations, or those of the publisher, the editors and the reviewers. Any product that may be evaluated in this article, or claim that may be made by its manufacturer, is not guaranteed or endorsed by the publisher.
